# Supplementary material for: Risk Factors for COVID-19 Morbidity and Mortality in Institutionalised Elderly People
Source: Int J Environ Res Public Health. 2021 Sep 28;18(19):10221. doi: 10.3390/ijerph181910221 (PMC8507792; doi:10.3390/ijerph181910221)
Supplement: Supplementary file 1 [file ijerph-18-10221-s001.zip › ijerph-1319464-supplementary.pdf]

Supplementary S1. Chronic treatments and infection by SARS-CoV-2.

	PCR COVID-19				
	[ALL]	Negative	Positive	Odds ratio	P value
	N=784	N=439	N=345		
ACE; ARB-II:					0.216
No	466 (59.4%)	252 (54.1%)	214 (45.9%)	Ref.	
Yes	318 (40.6%)	187 (58.8%)	131 (41.2%)	0.83 [0.62;1.10]	
ANTIPSYCHOTICS:					0.917
No	454 (57.9%)	253 (55.7%)	201 (44.3%)	Ref.	
Yes	330 (42.1%)	186 (56.4%)	144 (43.6%)	0.97 [0.73;1.30]	
ANTIDEPRESSANTS:					0.515
No	400 (51.0%)	229 (57.2%)	171 (42.8%)	Ref.	
Yes	384 (49.0%)	210 (54.7%)	174 (45.3%)	1.11 [0.84;1.47]	
ANTICHOLINERGICS:					0.222
No	763 (97.3%)	424 (55.6%)	339 (44.4%)	Ref.	
Yes	21 (2.68%)	15 (71.4%)	6 (28.6%)	0.51 [0.18;1.28]	
GABAERGICS:					0.435
No	758 (96.7%)	422 (55.7%)	336 (44.3%)	Ref.	
Yes	26 (3.32%)	17 (65.4%)	9 (34.6%)	0.67 [0.28;1.50]	
PROTON PUMP INHIBITORS					0.629
No	398 (50.8%)	219 (55.0%)	179 (45.0%)	Ref.	
Yes	386 (49.2%)	220 (57.0%)	166 (43.0%)	0.92 [0.70;1.22]	
NON-STEROIDAL ANTI-INFLA,MATORIES					0.347
No	757 (96.6%)	421 (55.6%)	336 (44.4%)	Ref.	
Yes	27 (3.44%)	18 (66.7%)	9 (33.3%)	0.63 [0.27;1.40]	
BENZODIAZEPINES:					0.685
No	471 (60.1%)	267 (56.7%)	204 (43.3%)	Ref.	
Yes	313 (39.9%)	172 (55.0%)	141 (45.0%)	1.07 [0.80;1.43]	
ANTIDIABETICS:					0.955
No	654 (83.4%)	367 (56.1%)	287 (43.9%)	Ref.	
Yes	130 (16.6%)	72 (55.4%)	58 (44.6%)	1.03 [0.70;1.50]	
STATINS:					0.097
No	604 (77.0%)	328 (54.3%)	276 (45.7%)	Ref.	

Yes	180 (23.0%)	111 (61.7%)	69 (38.3%)	0.74 [0.52;1.04]	
INHALERS:					0.890
No	664 (84.7%)	373 (56.2%)	291 (43.8%)	Ref.	
Yes	120 (15.3%)	66 (55.0%)	54 (45.0%)	1.05 [0.71;1.55]	
LEVODOPA:					0.222
No	763 (97.3%)	424 (55.6%)	339 (44.4%)	Ref.	
Yes	21 (2.68%)	15 (71.4%)	6 (28.6%)	0.51 [0.18;1.28]	
LEVOTHYROXINE					0.739
No	702 (89.5%)	395 (56.3%)	307 (43.7%)	Ref.	
Yes	82 (10.5%)	44 (53.7%)	38 (46.3%)	1.11 [0.70;1.76]	
OPIOIDS					0.401
No	676 (86.2%)	374 (55.3%)	302 (44.7%)	Ref.	
Yes	108 (13.8%)	65 (60.2%)	43 (39.8%)	0.82 [0.54;1.24]	
ANTIHYPERTENSIVES:					0.021
No	260 (33.2%)	130 (50.0%)	130 (50.0%)	Ref.	
Yes	524 (66.8%)	309 (59.0%)	215 (41.0%)	0.70 [0.52;0.94]	

Supplementary S2. Chronic drugs and mortality by SARS-CoV-2.

	[ALL]	No exitus	Exitus	Odds ratio	P value
	N=345	N=258	N=87		
ACE_ARB-II:					1.000
No	214 (62.0%)	160 (74.8%)	54 (25.2%)	Ref.	
Yes	131 (38.0%)	98 (74.8%)	33 (25.2%)	1.00 [0.60;1.64]	
ANTIPSYCHOTICS					0.293
No	201 (58.3%)	155 (77.1%)	46 (22.9%)	Ref.	
Yes	144 (41.7%)	103 (71.5%)	41 (28.5%)	1.34 [0.82;2.19]	
ANTIDEPRESSANTS					0.733
No	171 (49.6%)	126 (73.7%)	45 (26.3%)	Ref.	
Yes	174 (50.4%)	132 (75.9%)	42 (24.1%)	0.89 [0.55;1.45]	
ANTICHOLINERGICS:					0.171
No	339 (98.3%)	255 (75.2%)	84 (24.8%)	Ref.	
Yes	6 (1.74%)	3 (50.0%)	3 (50.0%)	3.02 [0.51;17.9]	
GABAERGICS:					0.459
No	336 (97.4%)	250 (74.4%)	86 (25.6%)	Ref.	
Yes	9 (2.61%)	8 (88.9%)	1 (11.1%)	0.41 [0.02;2.32]	
PROTON PUMP INHIBITORS					0.099
No	179 (51.9%)	141 (78.8%)	38 (21.2%)	Ref.	
Yes	166 (48.1%)	117 (70.5%)	49 (29.5%)	1.55 [0.95;2.54]	
NON-STEROIDAL ANTI-INFLAMMATORIES					0.238
No	336 (97.4%)	253 (75.3%)	83 (24.7%)	Ref.	
Yes	9 (2.61%)	5 (55.6%)	4 (44.4%)	2.45 [0.57;9.82]	
BENZODIAZEPINES:					0.127
No	204 (59.1%)	146 (71.6%)	58 (28.4%)	Ref.	
Yes	141 (40.9%)	112 (79.4%)	29 (20.6%)	0.65 [0.39;1.08]	
ANTIDIABETICS:					0.089
No	287 (83.2%)	209 (72.8%)	78 (27.2%)	Ref.	
Yes	58 (16.8%)	49 (84.5%)	9 (15.5%)	0.50 [0.22;1.02]	
STATINS:					0.337
No	276 (80.0%)	210 (76.1%)	66 (23.9%)	Ref.	
Yes	69 (20.0%)	48 (69.6%)	21 (30.4%)	1.39 [0.77;2.48]	

INHALERS:					0.521
No	291 (84.3%)	220 (75.6%)	71 (24.4%)	Ref.	
Yes	54 (15.7%)	38 (70.4%)	16 (29.6%)	1.31 [0.67;2.46]	
LEVODOPA:					0.171
No	339 (98.3%)	255 (75.2%)	84 (24.8%)	Ref.	
Yes	6 (1.74%)	3 (50.0%)	3 (50.0%)	3.02 [0.51;17.9]	
LEVOTHYROXINE					0.974
No	307 (89.0%)	229 (74.6%)	78 (25.4%)	Ref.	
Yes	38 (11.0%)	29 (76.3%)	9 (23.7%)	0.92 [0.39;1.97]	
OPIOIDS					0.210
No	302 (87.5%)	222 (73.5%)	80 (26.5%)	Ref.	
Yes	43 (12.5%)	36 (83.7%)	7 (16.3%)	0.55 [0.21;1.22]	
ANTIHYPERTENSIVES					1.000
No	130 (37.7%)	97 (74.6%)	33 (25.4%)	Ref.	
Yes	215 (62.3%)	161 (74.9%)	54 (25.1%)	0.98 [0.60;1.64]	

Supplementary S3. Acute treatment and mortality by SARS-CoV-2.

	[ALL]	No exitus	Exitus	Odd ratio	P value
	N=345	N=258	N=87		
TREATMENT WITH CORTICOIDS					0.181
No	284 (82.3%)	217 (76.4%)	67 (23.6%)	Ref.	
Yes	61 (17.7%)	41 (67.2%)	20 (32.8%)	1.58 [0.85;2.87]	
TYPES OF CORTICOIDS:					0.227
Prednisone	13 (21.3%)	11 (84.6%)	2 (15.4%)	Ref.	
Methylprednisolone	46 (75.4%)	28 (60.9%)	18 (39.1%)	. [.;.]	
Dexametasone	2 (3.28%)	2 (100%)	0 (0.00%)	. [.;.]	
